# Towards ecological psychiatry: The herbicide glyphosate disrupts behavior through microbiota-gut-brain axis

**DOI:** 10.1038/s41380-026-03693-2

**Published:** 2026-06-30

**Authors:** Rie Matsuzaki, Benjamin Valderrama, Gabriel S. S. Tofani, Soner Caner Cagun, Patrick Fitzgerald, Gerard Clarke, Eoin Gunnigle, John F. Cryan

**Affiliations:** 1 Department of Anatomy & Neuroscience, University College Cork, Cork, Ireland; 2 APC Microbiome Ireland, University College Cork, Cork, Ireland; 3 Department of Psychiatry & Neurobehavioral Sciences, University College Cork, Cork, Ireland

**Keywords:** Neuroscience, Molecular biology

## Abstract

Planetary and human health including mental health are closely interrelated. Increasing evidence also points to a role for the microbiota-gut-brain axis in maintaining optimum mental health. Emerging evidence raises concerns about the unintended toxicity of environmental exposure to xenobiotics, any substance foreign to the body, on the brain and behavior. Glyphosate is one of the most widely used active ingredients for herbicides for both agricultural and domestic applications. However, investigations on the effects of glyphosate at regulatory reference dose exposures on this axis are currently neglected.

Adult male and female mice were exposed to regulatory reference doses of glyphosate via drinking water for 7 weeks to assess its impact on gut microbiota composition, gut barrier function, physiology and behaviors including social interaction, anxiety, cognition, and the stress response. To establish causality, we conducted a microbiota transplantation examining whether behavioral phenotypes were phenocopied in naïve animals.

Regulatory reference dose glyphosate exposure primarily affected male mice, leading to impaired social novelty preference and increased anxiety-like behavior, whereas females exhibited a reduction in locomotor activity without other robust behavioral alterations. Transcriptomic analysis of the amygdala revealed gene expression changes consistent with observed behavioral deficits in males. Importantly, microbiota transfer from glyphosate-exposed donors selectively reproduced the social impairments in recipient mice, establishing the role of the glyphosate-remodeled microbiota in modifying social behavior.

These findings underscore the importance of evaluating ecologically relevant regulatory reference dose pesticide exposure and provide evidence that glyphosate impacts the microbiota-gut-brain axis to modify behavior. It further supports the concept that xenobiotics in the environment can impact mental health processes and further validates the concept of ecological psychiatry.

## Introduction

Accumulating evidence suggests that gut microbiota communicates with the brain, playing a crucial role in maintaining host physical and mental health [1–3]. This bidirectional communication, known as the microbiota-gut-brain axis, involves complex interactions that regulate various physiological processes, including mood, cognition and stress response [4, 5]. Disruptions in the microbiota-gut-brain axis have been linked to numerous psychiatric and neurological disorders [4, 6, 7]. This interplay can be disrupted by various factors, including diet, antibiotic medications, host genetics, delivery mode, and exposure to xenobiotics such as pesticides [8, 9]. Animal studies have highlighted the impact of exposure to various pesticides on both gut microbiota composition and brain functions, resulting in emotional and cognitive abnormalities [10].

Ecosystem and human health, including mental health, are closely interrelated, a perspective central to the “One Health” concept, which recognizes the interconnectedness of environmental, animal, and human health [11]. In psychiatry, this integrative view aligns with the framework of ecological psychiatry, which emphasizes how environmental factors influence mental health through biological mechanisms [12]. Within this context, environmental exposures are increasingly recognized as factors that can alter overall well-being and increase susceptibility to neuropsychiatric disorders [13, 14]. Herbicides, a class of pesticides specifically targeting unwanted vegetation, are widely used in agriculture and landscaping. Glyphosate, the active ingredient in the popular herbicide product Roundup®, is one of the most widely applied herbicides worldwide [15]. Glyphosate was long considered safe for mammals due to its mechanism of action on the shikimate pathway, which is absent in mammalian cells. However, growing in vitro, in vivo and limited epidemiological evidence suggest that glyphosate’s effects may not be limited to organisms with the shikimate pathway but could also extend to its host, such as humans [16, 17]. Recent research has also demonstrated that glyphosate not only crosses the blood-brain barrier but also upregulates pro-inflammatory markers and disrupts the brain transcriptome in a dose-dependent manner, indicating a potential for neurotoxicity in mice [18]. Consequently, glyphosate and glyphosate-based herbicides have been associated with impairments in locomotion, anxiety-like behaviors, depression-like behaviors, and social interactions in animal models such as mice [19–22] and zebrafish [23, 24].

Furthermore, an expanding literature suggests that glyphosate exposure alters host gut microbiota composition [19, 21, 25–28]. However, these studies have largely focused on taxonomic changes, often at high toxicological doses that do not reflect realistic environmental exposures. As a result, the functional relevance of the glyphosate-remodeled gut microbiota, particularly their contribution to behavioral outcomes, remains poorly understood [19, 26, 29]. Thus, the effects of glyphosate at regulatory doses, including levels considered safe, such as the acceptable daily intake (ADI) and no-observed-adverse-effect level (NOAEL), remain underexplored. Moreover, to establish causality between microbiota alterations and behavioral impairments, microbiota transplantation from pesticide-exposed donors to pesticide-naïve recipients offers a valuable approach in experimental animals [30, 31]. Despite its strength in testing causality, this technique has been rarely utilized in pesticide research on the microbiota–gut–brain axis [30], including studies involving glyphosate.

Taken together, we hypothesize that regulatory relevant dose of glyphosate exposure will alter gut microbiota composition, leading to behavioral impairments in mice. The causal impact of such effects will be investigated using microbiota transfer technique in animal models.

## Methods

### Animals

All animal experiments were conducted in accordance with approval from the Animal Experimentation Ethics Committee (AEEC) at University College Cork and the Health Products Regulatory Authority (HPRA), under project authorization number AE19130/P158 in accordance with the recommendations of the European Directive 2010/63/EU. All animals were assigned to either control or experimental groups randomly and each animal experiments were conducted once. For the initial study, C57BL/6 mice (Envigo Laboratories, UK; 7.5–8.5 weeks of age at the beginning of glyphosate treatment) were used (*n* = 11/treatment/sex, except for one loss in male control and female low-dose groups). For the microbiota transplantation study, a separate cohort of male C57BL/6 mice (Envigo Laboratories, UK; 8 – 8.5 weeks of age at the beginning of treatment) was used, comprising of donor and recipient groups (*n* = 23 total per cohort; *n* = 11 control, *n* = 12 experimental). Minor variation in final group sizes resulted from the loss of one animal during habituation. Water (with or without glyphosate) was given *ad libitum* throughout the experiment. Researchers were blinded to treatment allocation during behavioral testing, tissue collection, and molecular analysis.

### Glyphosate administration

**Fig. 1:**
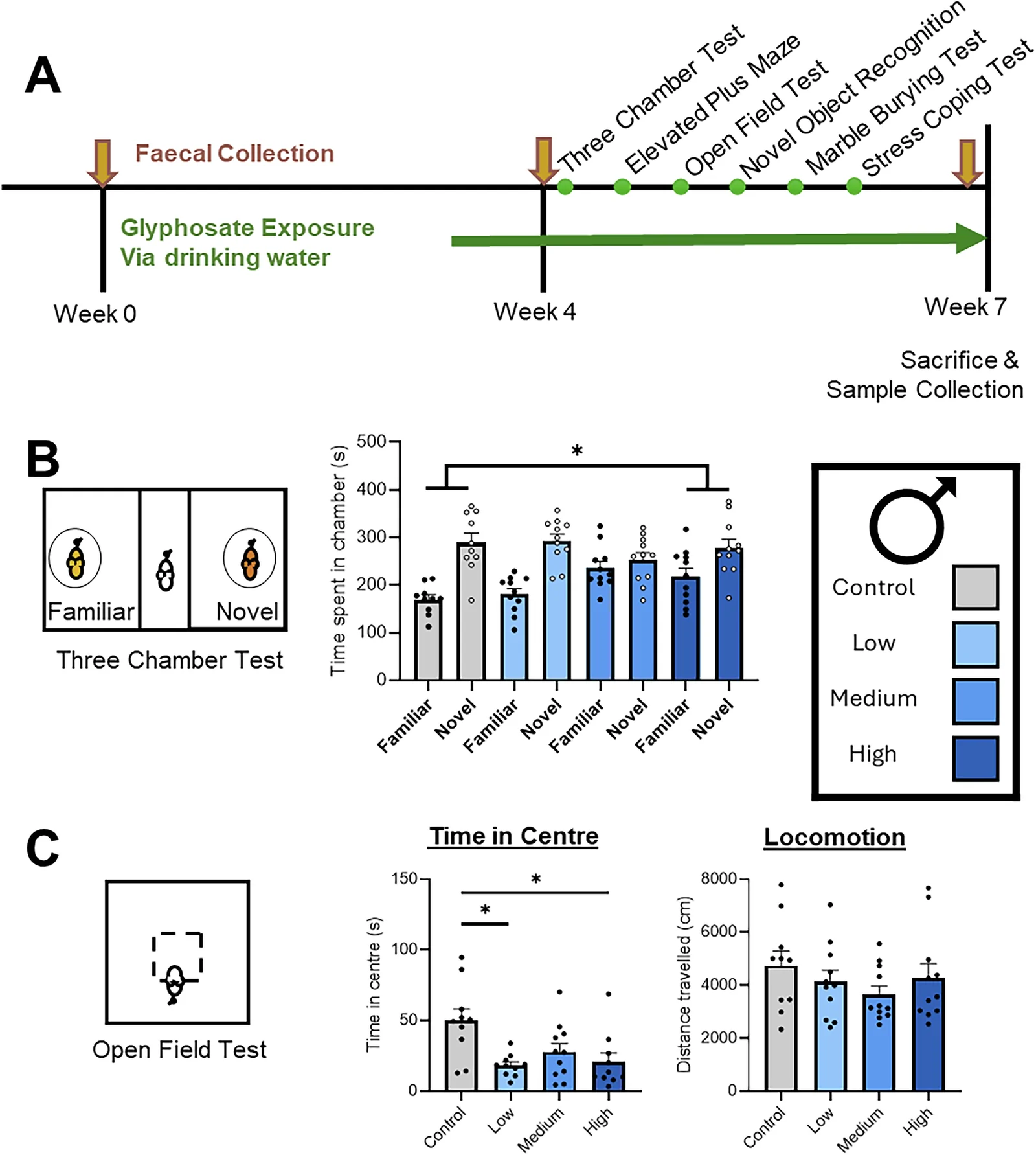
Glyphosate exposure disrupted social cognition and increased anxiety-like behavior in males. (**A**) Experimental timeline of adulthood glyphosate exposure (**B**) Social novelty preference behavior (Two-way repeated-measures ANOVA) (**C**) Anxiety-like behavior and locomotion (Kruskal-Wallis test followed by pairwise comparisons) *p ≤ 0.05; *n* = 10–11 per treatment group per sex; data represent mean ± SEM.

The experimental design is presented in Fig. 1A (Sex-comparison) and Fig. 4A (Microbiota Transplantation). For the former, glyphosate (Sigma-Aldrich) was administered via drinking water (0, 0.5, 5, 50 mg/kg/day) for 7 weeks. The exposure period was selected based on previous studies reporting detectable microbial alterations [32]. For the follow-up, cecal microbiota transplant, the donor received drinking water either with or without glyphosate (50 mg/kg/day) for 4.5 weeks. The lowest dose tested corresponded to the established acceptable daily intake (ADI) for glyphosate, which is derived from the NOAEL as defined by the European Commission and the European Food Safety Authority, while the medium and high doses represented 10- and 100-fold multiples of the ADI, respectively. For the recipient cohort, mice were randomly assigned to a specific donor and received their respective microbiota transplant according to the schedule shown in Fig. 4A.

### Behavioral test batteries

A series of behavioral tests were used to assess different aspects of behavior (Fig. 1A). Detailed behavioral apparatus and analysis parameters could be found in the supplement. The following behaviors were studied:

- Three-chamber test (3CT, social): Mice were placed in a rectangular arena divided by panels into three chambers. Wire mesh cages were placed in the side chambers, which contained a combination of a familiar mice and either an inanimate object (sociability phase) or a novel mouse (social novelty phase). The experimental mice freely explored the arena for ten minutes per phase.
- Elevated plus maze (EPM, anxiety): Mice were placed in the center of a plus-shaped maze elevated 1 m above the ground. The mice freely explored the whole arena, including the open and closed arm, for 5 min.
- Open field test (OFT, anxiety): Mice were placed in a square arena and freely explored for 10 min.
- Marble burying test (stereotypical behavior): Mice were placed in a cage, filled with sawdust bedding and 20 marbles placed on top. The mice freely explored the arena and buried the marbles for 30 min (males) or 15 min (females).
- Stress coping test (stress): Mice were placed in a clear glass cylinder filled with water and their swimming behavior was recorded for 6 min.

### Corticosterone measurement

Tail blood samples were collected prior to and every 30 min until 2 h following the stress coping test to measure the corticosterone response following the acute stress. Plasma corticosterone was analyzed using ELISA kit (Enzo Life Sciences, Belgium) following the manufacturer’s protocol.

### Cecal microbiota transplantation

Cecal content was homogenized in sterile PBS with 20% glycerol (w/v) and filtered through a 70-μm strainer to create a 100 mg/ml cecal slurry, then stored at −80 °C until use.

Recipient mice were given an antibiotic cocktail (Ampicillin 1000 mg/L, Vancomycin 500 mg/L, Imipenem 250 mg/L, and Gentamicin 1000 mg/L; Discovery Fine Chemicals, UK) in their drinking water for one week to deplete existing gut microbiomes and to facilitate engraftment of donor-derived microbiota. Following antibiotic treatment, recipient mice were gavaged with microbiota from either glyphosate-exposed or control donors. This control design allows comparison between glyphosate-modified and unexposed microbiota, isolating the effect of glyphosate exposure. The solution was freshly prepared daily and changed every other day. For transplant, 100 µl of inoculum was orally gavaged over three consecutive days initially, followed by two booster doses weekly, totaling eight doses.

### RNA & DNA extraction

Total RNA was extracted from ileum, distal colon and left amygdala using the mirVana™ miRNA Isolation kit (Invitrogen^TM^, ThermoFisher Scientific, UK) following the manufacturer’s protocol.

For the initial study, DNA was extracted from endpoint feces; for the microbiota transplantation study, DNA was extracted from cecal microbiota transplant inoculums (donor) and endpoint cecal content (recipient). Total DNA was extracted using QiaAMP Power Fecal Pro kit (QIAGEN, Germany) following the manufacturer’s protocol, with the addition of an initial bead-beating step.

Isolated nucleic acids were kept at −80 °C until further use. For DNA sequencing, samples were outsourced to Teagasc Next Generation DNA sequencing Facility.

### Quantitative real-time polymerase chain reaction

For qRT-PCR, complementary DNA was synthesized from extracted RNA using Applied Biosystems™ High Capacity cDNA kit (Applied Biosystems, Warrington, UK) according to the manufacturer’s instructions. Then, this cDNA was used for SYBR® Green PCR Master Mix (Applied Biosystems) to perform qRT-PCR. Genes were selected based on their role in gut barrier functions, with *Gapdh* (ileum and colon) and *ActinB* (recipient, left amygdala) as housekeeping genes. Gene expression levels were then analyzed using a Lightcycler 480 II (Roche). Gene expression was analyzed by transforming the cycle threshold value (Ct) using the 2−ΔΔCT method [33].

### Transcriptomic analysis

In brief, mRNA sequencing was performed on microdissected amygdala, encompassing the major amygdalar subnuclei, using Illumina NovaSeq, aligned to the *Mus musculus* reference genome (GRCm38) with quality assessed with FastQC. Gene annotation was done using kallisto [34], and downstream analysis was conducted in Rstudio. Permutational Multivariate Analysis of Variance (PERMANOVA) was used on centered log-ratio (CLR) transformed data to explore overall variation in gene expression, and targeted differential gene expression analysis focused on genes encoding the pathways “social behavior” and “learning or memory” (GO terms: GO:0035176, GO:0007611) without false discovery rate (FDR) correction, given the targeted scope.

### Gut microbiome profiling using whole genome sequencing

Briefly, FASTQ files were quality-checked using Kneaddata v0.12.0, followed by decontamination with Bowtie2 v2.5.1, aligning against the GRCm39 mouse genome as a reference (NCBI RefSeq assembly: GCF_000001635.27). The remaining reads were then aligned to bacterial genomes from the web of life database (release 1). Taxonomic and functional profiles were generated with Woltka v0.1.3, and functional annotations were refined with Omixer-rpmR to identify Gut-Brain Modules (GBMs).

### Gut microbiome profiling using 16S sequencing

In brief, FASTQ files were processed in R (v4.1.1) using DADA2 (v1.22.0) to construct an amplicon sequence variant (ASV) count table up to the genus level, with custom parameters for filterAndTrim. Taxonomic assignment was conducted using the SILVA database (v138). Functional inference of genomic content in terms of Enzyme Commission (EC) numbers was performed with PICRUSt2 (v2.4.1) to annotate GBMs.

### Gut microbiome analysis

Statistical analyses were conducted in R (v4.2.0) and RStudio (v2022.7.1.554). Alpha diversity metrics were calculated using the microbiome package (v1.24.0) and assessed with linear models. CLR transformation was applied via the decostand function (vegan v2.6.4), and principal component analysis (PCA) was performed using Euclidean distances on CLR-transformed data (Aitchison distance). Beta diversity differences were evaluated using PERMANOVA (10,000 permutations) in the adonis2 function. Differential abundance of taxa and GBMs was assessed with general linear models on CLR-transformed tables, with an FDR threshold of 0.1 (Benjamini-Hochberg). Plots were generated using ggplot2 and patchwork packages.

### Other statistical analysis

Statistical analyses were designed to assess the effects of each glyphosate dose relative to the control group. Direct comparisons between different glyphosate doses were not performed, as the study was not designed to evaluate dose–response relationships across exposure levels. Each outcome domain was analyzed independently with pre-specified hypotheses, and no additional multiplicity correction across domains was applied. All statistical analyses were conducted using SPSS 28 (IBM, USA). Technical outliers were removed before commencing the statistical analysis. Grubbs outlier test was used to exclude a maximum of one outlier per group. Prior to statistical analysis, normality was tested using the Shapiro–Wilk test and homogeneity of variances using Levene’s test. Nonparametric data were analyzed using the Kruskal–Wallis test followed by post-hoc Dunn’s tests. All data are represented as mean ± SEM. All statistical tests were two-sided, with statistical significance set at *p* ≤ 0.05. Changes in body weight and daily water intake over the course of the experiment, as well as corticosterone levels, were analyzed using a repeated measures general linear model. For 3CT, two-way repeated-measures ANOVA was used. For all other measures, one-way ANOVA (male and female study) and independent t-test (microbiota transplantation study) were conducted.

All details for general housing, behavioral tests, sample collection, corticosterone measurement, genomic sequencing, transcriptomic analysis, gut microbiome profiling and analysis can be found in the supplement.

## Results

Below the main results are described; all other detailed results and statistical analysis can be found in the supplement and/or figure legend.

### Glyphosate exposure had no significant effects on overt physiology

Glyphosate exposure was relatively well tolerated by both male and female mice, while females showed a minor difference in water drinking levels (Overall: *F*_3,9_ = 5.43, *p* = 0.021; High: *p* = 0.023; Figure S1B). There were no treatment-dependent differences in body weight gain (Figure S1A) or defecation patterns during behavioral tests in either sex (data not shown). In males, glyphosate exposure was associated with minor changes in organ weights, such as reduced spleen size at the medium dose (Overall: *F*_3,42_ = 4.11, *p* = 0.013, Medium: *p* = 0.008; Figure S2), an effect absent in females. Plasma corticosterone levels, measured after stress coping test, showed no differences in physiological stress responses across treatment groups for either sex (Figure S3).

### Glyphosate exposure affects behavior and gut microbiota

We first investigated the impact of regulatory reference dose-range glyphosate exposure on behavior, gut microbiota and transcriptomic profiles in both male and female mice (Fig. 1A). For clarity, results are presented separately for male and female subjects and male findings are presented first. In the 3CT, during the sociability phase, mice prefer to interact with a conspecific mouse over an inanimate object, which we observed for all groups in males (Figure S4A). During the social novelty phase, mice exhibit a preference for novel over familiar stimuli. In males, this preference was observed in every treatment group except the high-dose (Chamber*treatment: *F*_*3,39*_ = 3.073, *p* = 0.039, *η*^*2*^*p* = 0.19; High: *p* = 0.049), indicating a disrupted social novelty preference due to glyphosate exposure (Fig. 1B).

In the OFT, the amount of time spent in the center significantly decreased among male mice in a dose-specific manner (Overall: *H*_3_ = 10.97, *p* = 0.012, *η*^*2*^ = 0.22), post-hoc analysis showing heightened anxiety levels for low and high treatment groups (Low: *p* = 0.041; High: *p* = 0.016; Fig. 1C). Although not statistically significant, visual inspection also supports that this decrease is also recorded for the medium dose. However, no significant impact on anxiety-like behavior was observed in the EPM, as indicated by the open arm ratio (Figure S4B). No other exposure dependent behavioral impairments were observed (Figure S4C, D).

**Fig. 2:**
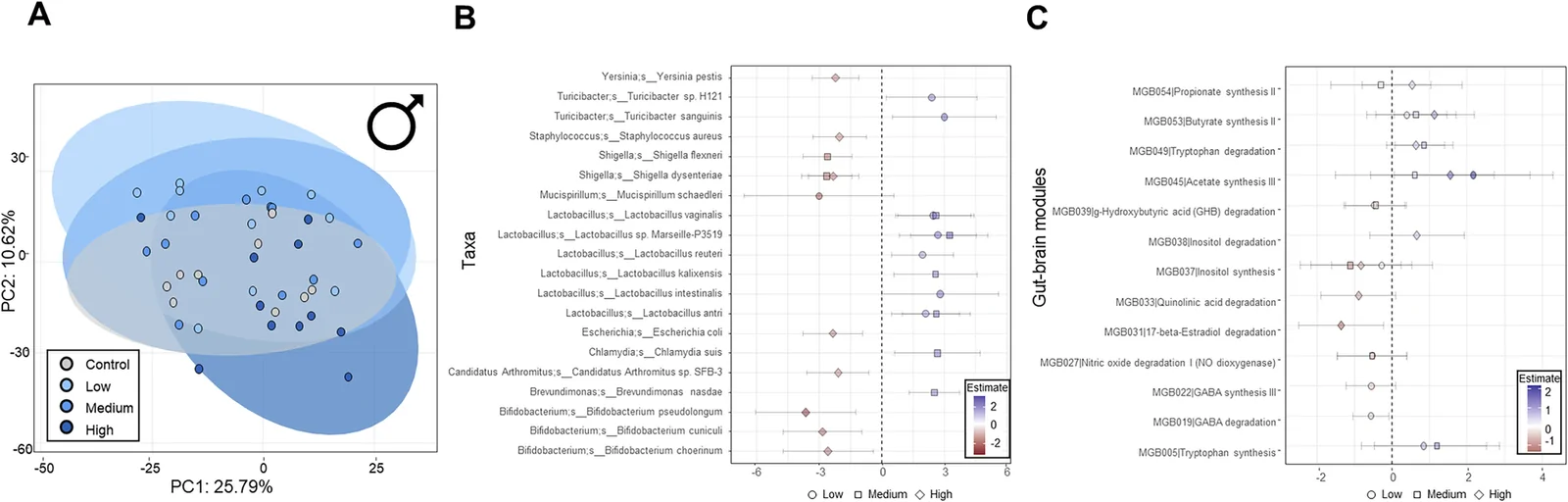
Glyphosate exposure shifted the gut microbiota community structure while had a minor impact on differentially abundant taxa and gut-brain modules on the male mice. (**A**) Principal component analysis of beta diversity (Ellipse denotes 95% CI) (**B**) Differential abundance analysis of bacterial taxa(General linear model, Beta estimate ± 95% CI) (**C**) Differential analysis of predicted gut–brain modules (General linear model, Beta estimate ± 95% CI). For figure B and C, only the top eight taxa per dose exhibiting the largest absolute beta estimates are displayed for clarity; taxa not among the top eight for a given dose are not shown. *n* = 10–11 per treatment group.

Glyphosate exposure exerted subtle effects on the male gut microbiota. By fitting linear models to analyze alpha diversity, which reflect the diversity within a community, showed no significant differences across treatment male groups (Figure S5). Conversely, beta diversity, reflecting between-group differences in community composition, was significantly altered following exposure as indicated by the PERMANOVA analysis (Pseudo *F*_*3,41*_ = 1.55, *p* < 0.001, 10000 permutations, Fig. 2A). Despite the absence of significant differences in taxa abundance, we investigated taxa with a larger degree of change when compared to the control group, as indicated by the absolute value of the beta estimate in the linear models. Notably, the direction of change was consistent across dosages: an increase in several *Lactobacillus* strains (low and medium groups) and a decrease in *Shigella dysenteriae* (medium and high groups). High-dose exposure also resulted in a reduction of *Bifidobacterium* strains (Fig. 2B). Predicted functional analyses of GBMs showed no significant differences in the neuroactive potential upon exposure. However, interestingly, all doses exhibited an increase in butyrate synthesis II and acetate synthesis III, alongside a decrease in inositol synthesis (Fig. 2C). Given the close relationship between gut barrier function and gut microbiota, gene expression related to the gut barrier was assessed, revealing no significant changes in the analyzed genes (Figure S6).

Considering the behavioral alterations noted in males, we hypothesized that transcriptional differences would occur in the brain. Transcriptomic analysis of the amygdala revealed subtle impacts of glyphosate exposure. Globally, no group differences were observed in PCA (*F*_*3,42*_ = 0.75, *p* = 0.94, *R²* = 0.05; Figure S7A). Given that we observed changes in social cognition, we decided to take a targeted approach and filter the transcriptome for genes associated with “social behavior” and “learning or memory” as described in the Gene Ontology database [35]. After filtering for genes relevant to our behavioral phenotype, we identified alterations in the regulation of 2, 3, and 13 genes in the low, medium, and high-dose groups, respectively, indicating that the high dose concentrated most of the alterations (Figure S7).

**Fig. 3:**
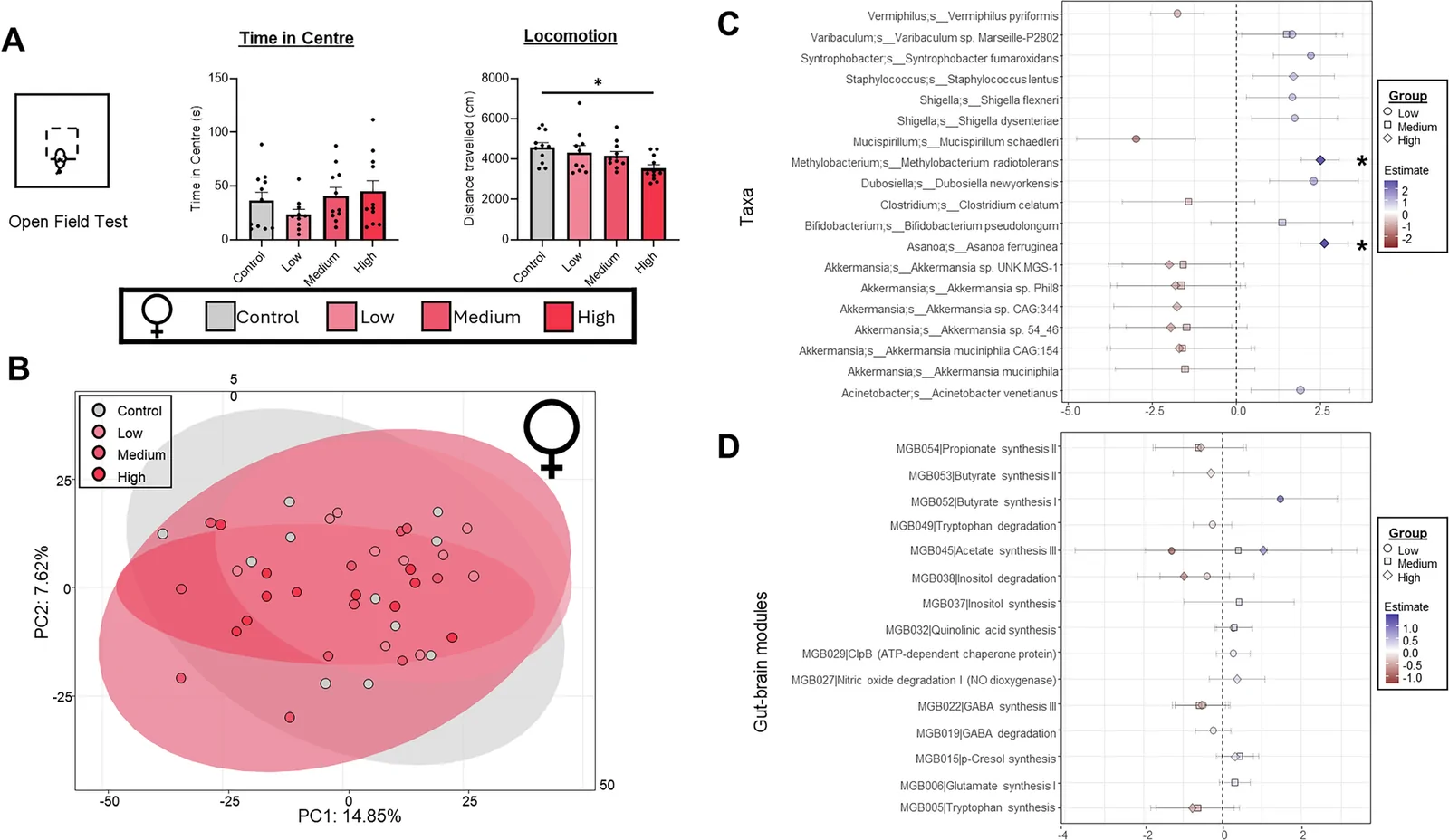
Glyphosate exposure exhibited decreased locomotion with changes in the gut microbiota in females. (**A**) Anxiety-like behavior and locomotion (one-way ANOVA followed by post-hoc Tukey analysis; *p ≤ 0.05; mean ± SEM) (**B**) Principal component analysis of beta diversity (Ellipse denotes 95% CI) (**C**) Differential abundance analysis of bacterial taxa (General linear model, Beta estimate ± 95% CI) (**D**) Differential analysis of predicted gut–brain modules (General linear model, Beta estimate ± 95% CI). For figure C and D, only the top eight taxa per dose exhibiting the largest absolute beta estimates are displayed for clarity; taxa not among the top eight for a given dose are not shown. *n* = 10–11 per treatment group.

Regarding the female mice, we observed fewer behavioral impacts in females following glyphosate exposure, notably a decrease in locomotion only in the high-dose group (Overall: *F*_*3,42*_ = 3.44, *p* = 0.026, *η*^*2*^ = 0.02; High: *p* = 0.019; Fig. 3A). Unlike males, we recorded no other behavioral disturbances including social novelty preference and anxiety-like behavior (Fig. 3A, S4); however, interpretation is limited as control animals did not display the expected baseline preference, preventing conclusive assessment.

Similar to males, glyphosate exposure in females resulted in minimal impacts on the gut microbiota. Alpha diversity showed no significant changes (Figure S5), while beta diversity analyses indicated a significant shift in the bacterial community composition post-exposure (Pseudo *F*_*3,42*_ = 1.44, *p* < 0.01, 10000 permutations; Fig. 3B). Notably, two strains (*Methylobacterium radiotolerans* and *Asanoa ferruginea*) significantly increased in abundance at high doses when compared to the female control group. Although not statistically significant, a consistent decrease in taxa belonging to the genus *Akkermansia* across medium and high doses was observed (Fig. 3C). Functional analysis of GBMs revealed no significant alterations. As with males, no significant changes were observed in gene expression related to gut barrier function (Figure S6).

Overall, glyphosate exposure resulted in more pronounced behavioral impairments in males compared to females, whereas females displayed more characteristic alterations in gut microbiota composition. These findings suggest that males and females respond differently to glyphosate exposure.

### Social behavioral impairment, but not anxiety-like behavior, was transferred via microbiota transplantation

**Fig. 4:**
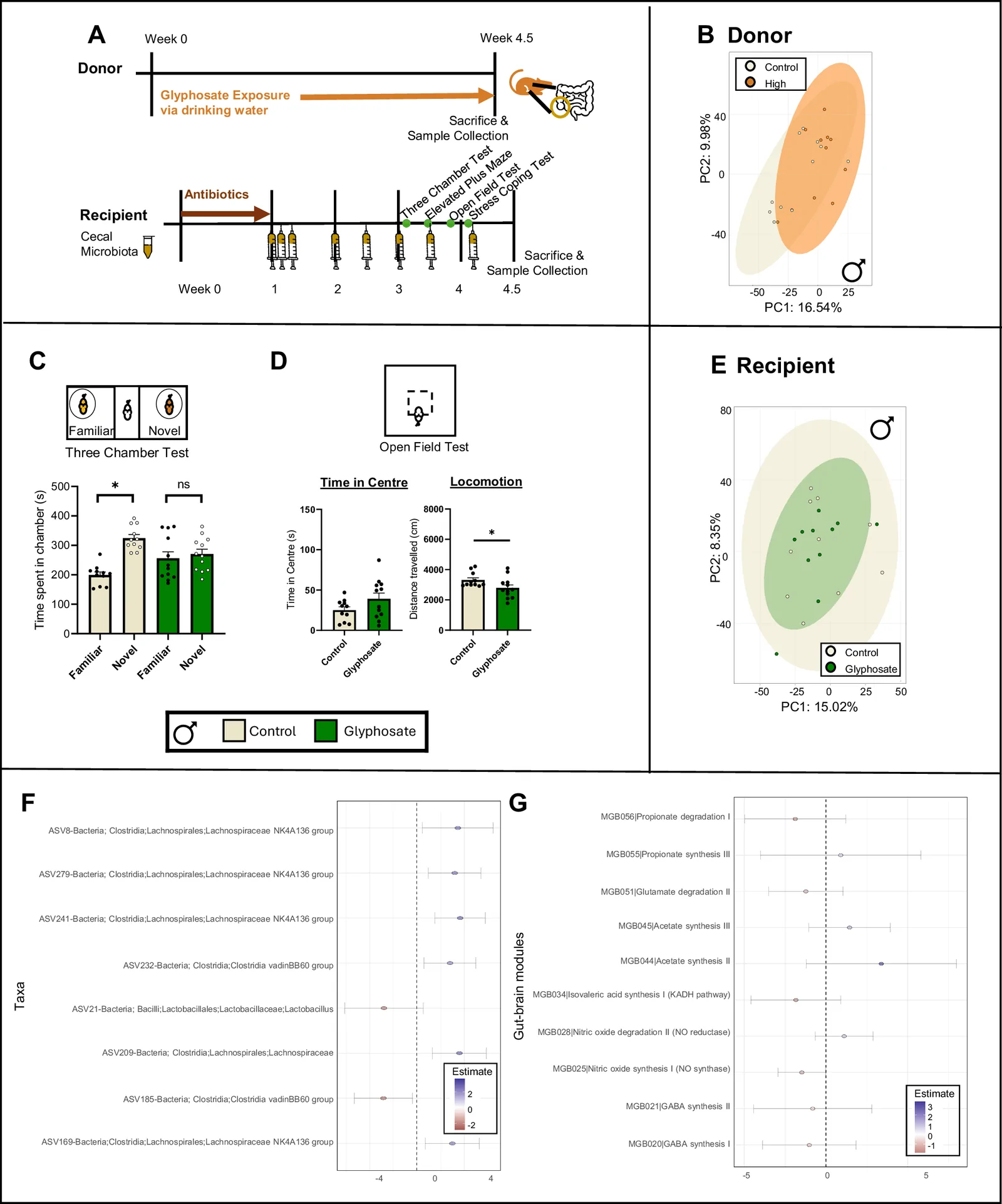
Glyphosate remodeled gut microbiota phenocopied domain-specific behavior in males. (**A**) Experimental timeline for cecal microbiota transplantation (**B**) Principal component analysis of donor beta diversity (Ellipse denotes 95% CI) (**C**) Social novelty preference behavior (Two-way repeated-measures ANOVA; *p ≤ 0.05; mean ± SEM) (**D**) Anxiety-like behavior and locomotion (t-test; *p ≤ 0.05; mean ± SEM) (**E**) Principal component analysis of recipient beta diversity (Ellipse denotes 95% CI) (**F**) Differential abundance analysis of bacterial taxa (General linear model, Beta estimate ± 95% CI) (**G**) Differential analysis of predicted gut–brain modules (General linear model, Beta estimate ± 95% CI). For figure F and G, only the top eight taxa per dose exhibiting the largest absolute beta estimates are displayed for clarity; taxa not among the top eight for a given dose are not shown. *n* = 11–12 per treatment group.

To further investigate the underlying causes of social behavioral impairment following glyphosate exposure, in males, we conducted a cecal microbiota transplant to glyphosate-naïve mice to assess whether microbiota is the primary driver of observed behavioral changes (Fig. 4A). No difference in body weight gain, organ weights or plasma corticosterone levels were observed (Figure S8A, B, F).

After confirming the cecal microbial shift in the donor (*F*_*1,22*_ = 1.72, *p* < 0.01, *R*^2^ = 0.076; Fig. 4B), we examined whether behavioral phenotypes were transferred to the recipients. The 3CT, tested following 2 weeks of microbiota transfer, revealed that the disruption of social novelty preference persisted in glyphosate microbiota recipients compared to the control microbiota recipients (Chamber*treatment: *F*_*3,21*_ = 6.32, *p* = 0.020, *η*^*2*^*p* = 0.23, Fig. 4C). In the OFT, there was no evidence of a transfer of anxiety-like behavior from the donor to the recipient (Fig. 4D, left). However, glyphosate microbiota recipients demonstrated reduced locomotion compared to the control group (Fig. 4D, right). No other behavioral differences were observed upon glyphosate exposure (Figure S8C, D, E). Moreover, the changes in amygdalar gene expression, seen upon direct glyphosate exposure earlier, were not replicated in the glyphosate microbiota recipients (Figure S8G). While major changes were not observed, microbiota transplantation from glyphosate-exposed donors revealed subtle trends in gut microbial composition and function in recipients. First, no significant alterations were observed in any alpha diversity indices, although the Chao 1 index approached significance for glyphosate-exposed donors compared to controls (*p* = 0.054; Figure S9). Second, microbiota community shifts were not detected in the cecal content of the recipients at the end of the experiment (*F*_*1,22*_ = 1.06, *p* = 0.267, *R*^2^ = 0.048; Fig. 4E). Third, no significant differences in taxa abundance were observed in the recipients (Fig. 4F). Furthermore, predicted functional analyses revealed no significant alterations (Fig. 4G).

Taken together, the microbiota transplantation from glyphosate-exposed donors was sufficient to transfer only social behavioral impairments to glyphosate-naïve recipients.

## Discussion

While some pesticides with neuroactive modes of action have been studied in relation to the microbiota–gut–brain axis, the causal contribution of the microbial changes to behavioral alterations remains underexplored, even for widely used compounds such as glyphosate. To this end, we addressed this knowledge gap by investigating the effects of regulatory reference range of glyphosate exposure on various aspects of the microbiota-gut-brain axis in mice. Our findings revealed that exposure to glyphosate at tested doses is sufficient to disrupt behavior and shift the gut microbial community. In male mice, behavioral alterations were observed alongside corresponding changes in brain transcriptomic profiles and shifts in gut microbial community structure. Female mice similarly exhibited community-level microbial shift, with additional alterations at an individual taxa level. Crucially, by conducting a microbiota transplantation experiment, in male mice, we confirmed that the glyphosate-remodeled microbiota specifically mediated the social behavioral disturbances, but not anxiety-like phenotypes, suggesting a primary role of the gut microbiota in these effects. Together, these results position the gut microbiota as a mechanistic link between environmentally relevant glyphosate exposure and altered social behavior, complementing prior association studies and extending them by demonstrating causality.

### Glyphosate-remodeled gut microbiota modifies social behavioral deficits

Our study builds on previous findings, demonstrating that both gut microbiota and behavior are impacted by glyphosate exposure [19, 21, 29]. Notably, glyphosate exposure was associated with a reproducible shift in overall gut microbial community structure, despite limited changes at the level of individual taxa across the dose range tested here. This observation suggests that community-level reorganization, rather than discrete taxon-specific alterations, may be sufficient to influence behavioral outcomes. Such effects may arise from emergent community interactions, including cross-protection and cross-sensitization among microbial members, whereby the collective metabolic and functional capacity of the microbiota is altered. Recent work using defined microbial communities has shown that community resilience diminishes as dose increases, leading to more pronounced taxon-level disruptions at higher doses [36], whereas lower-dose exposures, which we examined here, may preferentially manifest only as a community-level shifts. Importantly, shifts in community structure can result in loss or gain of function at the ecosystem level, even in the absence of marked taxonomic depletion [37]. Therefore, these findings collectively suggest the notion that community-level reorganization may be influencing host neurobehavioral processes.

Consistent within this framework, several studies employing glyphosate doses approaching the NOAEL have reported more pronounced taxa-specific alterations [19, 21, 38]. In contrast, a study with similar low-range doses to those used here observed taxa level alterations only following a longer exposure period [25], suggesting that exposure duration may be a critical determinant of species-level disruption. Future studies employing longer-duration, low-dose exposure paradigms will be essential to delineate the temporal evolution of microbiota community reorganization and to determine whether sustained exposure is necessary to elicit more pronounced, taxon-level changes that contribute to gut–brain axis signaling.

To further elucidate our findings, we examined taxa that exhibited the strongest alterations, as indicated by beta estimates, which reflect the magnitude and direction of the association with glyphosate exposure. Although these changes did not reach statistical significance, they suggest modest trends, in males, such as increases in*Lactobacillus* spp. and decreases in *Bifidobacterium*, consistent with previous findings [19, 25, 39]. Both taxa are known producers of short-chain fatty acids (SCFAs), microbial metabolites crucial for healthy microbiota–gut–brain communication [40–42]. While speculative, these trends may provide insights into potential functional shifts arising from community-level reorganization, rather than from discrete sensitivities of individual taxa to glyphosate. For instance, certain *Lactobacillus* strains exhibit relative resistance, possibly due to variations in the structure of the shikimate pathway enzyme that determines glyphosate sensitivity [43], whereas *Bifidobacterium* may be more susceptible [44]. Even in the absence of overt taxonomic depletion, such community-level changes could influence SCFA production, mucosal immunity, or intestinal morphology, highlighting the importance of evaluating microbiota function and host interactions at the community rather than individual taxon level. Moreover, some bacterial strains are capable of metabolizing glyphosate into its breakdown products, such as AMPA and sarcosine [45]. Although the extent to which this occurs in vivo remains unknown, AMPA has been reported to elicit behavioral effects comparable to glyphosate [46, 47], suggesting that future investigations examining both glyphosate and its metabolites may help clarify their respective roles in shaping gut–brain interactions.

Similarly, gut-brain modules, which serve as a functional surrogate of microbiota neuroactive potential [48–50], showed only modest, non-significant changes, with wide confidence intervals suggesting that the doses tested may not be sufficient to majorly disrupt gut microbiota functions per se. Nonetheless, exploratory trends were observed in SCFA-related pathways—particularly acetate, butyrate, and propionate—aligning with previous findings [25, 51]. While these patterns are not statistically robust, they should not be interpreted as reassuring; even modest functional disruptions may arise from community-level reorganization and have profound consequences for host neurobehavioral processes. It may be worth noting that since GBMs are inferred from taxonomic composition, they may underestimate the direct effects of community-level changes. Microbiota transfer studies, complemented by approaches such as gnotobiotic models and metabolomic profiling, and direct assessments of mucosal immunity or intestinal morphology, can help reveal whether such community-level alterations translate into measurable downstream effects on host health.

While an increasing body of research has implicated glyphosate in altering both behavior and microbiota, the causal relationship between the two remains unexplored. To our knowledge, this is the first study to confirm the domain-specific phenocopy of behavioral disruptions through cecal microbiota transplantation from glyphosate-exposed mice to glyphosate-naïve recipients. In our study, male donors exposed to a single dose of glyphosate were used to model this relationship. The highest dose tested, 50 mg/kg, was selected due to its significant behavioral, microbial, and transcriptomic alterations observed in the original study in males. Our findings demonstrated that glyphosate-remodeled microbiota were able to replicate impairments in social cognition, but not anxiety-like behavior, suggesting a stronger association with social behavioral deficits. This is not surprising, as social behavior has been strongly linked to the gut microbiota [52]. Changes in social behavior reflect underlying neuronal alterations—such as in synaptic plasticity or neuroimmune signaling—though further clarification is needed to determine whether these effects are primarily mediated by microbiota shifts. On a broader scale, additional research is needed to determine whether the observed behavioral impairments are driven by subtle bacterial alterations or by other gut microbiota components, such as archaea, viruses, and eukaryotes [53].

We hypothesized that subsequent alterations in brain transcriptomics could be contributing to the observed behavioral changes. The amygdala was selected for analysis given its central role in regulating anxiety-related behaviors and social processing. Our targeted analysis revealed a dose-dependent increase in learning-related genes, suggesting a potential link to underlying genetic modulation. Particularly, the increase in beta-2 microglobulin (*B2m*) stands out, as previous studies have reported a correlation between B2M levels and urinary glyphosate concentrations in both farmers and individuals following accidental ingestion [54, 55]. Elevated B2M expression has also been previously observed in mice [56, 57]. Elevated B2M levels in the brain have been associated with cognitive decline [58], which may help explain the impaired social novelty preference observed in our study. Additionally, the downregulation of the activity-regulated cytoskeletal-associated protein gene (*Arc*), a key gene involved in long-term memory, aligns with prior research linking Arc deficiency to deficits in social novelty in mice [59]. While other regions such as the hippocampus, striatum and prefrontal cortex are also relevant, comprehensive multi-region transcriptomic profiling was beyond the scope of the present study and represents an important direction for future work. Notably, given the lack of replication upon microbiota transfer, these molecular changes may not be the primary drivers of behavioral alterations. Instead, our results are consistent with the possibility that behavioral and molecular effects may arise through distinct, potentially independent pathways, underscoring the need for future studies to investigate alternative mechanisms, such as peripheral immune signaling, or metabolite-mediated gut–brain communication, that could contribute to glyphosate-induced behavioral outcomes [10].

### Influence of biological sex on behavioral and microbial responses to glyphosate exposure

Males and females were analyzed separately to account for biological variability; however, the study was not designed for direct sex comparisons. Accordingly, sex-specific observations are discussed descriptively and interpreted within the biological context of each sex rather than as direct comparisons. Under these conditions, glyphosate exposure produced distinct response patterns across sexes. In females, glyphosate exposure was associated with changes in gut microbiota composition including increased abundance of environmental-associated taxa such as *Methylobacterium radiotolerans* and *Asanoa ferruginea*, along with reductions in *Akkermansia*-related strains—a genus involved in mucin degradation and SCFA production [60]. Such observations may be influenced by intrinsic sex-related differences in gut microbiota composition, shaped by factors such as hormones and immunity, which are well documented [61]. Consistent with this interpretation, previous studies have reported sex-dependent effects of pesticide exposure on gut microbiota composition [62, 63], including females exhibiting more pronounced microbial alterations in response to glyphosate-based herbicides in rodent models [21, 27]. Although direct microbiota comparisons between sexes were not performed here, these baseline differences may contribute to divergent microbial and host responses to toxicant exposure.

While these microbial changes observed in were not accompanied by robust behavioral deficits comparable to those observed in males, previous studies have similarly reported that glyphosate-based herbicide exposure affects sociability in male but not female mice [21]. In the present study, interpretation of female social behavior outcomes is further constrained by the absence of a clear social novelty preference in control females, which limits direct inference regarding exposure-related effects in this behavioral domain. This may in part reflect variability associated with the estrous cycle, which is known to influence female social behavior [64]. Together, these findings underscore the importance of considering biological sex as a contextual factor that may modulate microbiota–host interactions. Future studies specifically designed to examine sex-specific toxic response will be essential to better understand how biological sex shapes gut–brain axis responses to environmental exposures.

One of the limitations of our study is the lack of synchronization of the estrous cycle in female mice, which may have introduced variability in social behavior measures. However, other tested behaviors generally appear robust to estrous variability [65]. In addition, although antibiotic-mediated microbiota depletion is commonly used to facilitate donor microbiota engraftment, such treatment may itself influence host physiology [31]. Importantly, all recipient groups underwent identical antibiotic treatment, minimizing potential confounding effects. Future studies could further refine causal inference by incorporating baseline microbiota transplantation or protocols without prior depletion. Finally, while the exposure paradigm was designed to reflect regulatory-relevant dosing, further research is needed to determine how these findings translate to real-world exposure scenarios, including variations in application patterns, environmental concentrations, and exposure chronicity. Glyphosate is detectable in surface and drinking water, typically at concentrations below those tested here, although occasional environmental measurements approach similar levels [66, 67]. Moreover, although drinking water was used as the exposure route in this study, dietary intake is considered a primary route of human exposure [67], which may alter toxicokinetics and interactions with the gut microbiota. Accordingly, caution is warranted when extrapolating these findings to human health, given interspecies differences in physiology, metabolism, dose conversion and exposure patterns [68].

### Conclusions: towards ecological psychiatry

In summary, our findings demonstrate that regulatory-relevant doses of glyphosate exposure can lead to behavioral impairments in non-target species through the microbiota-gut-brain axis—behavioral-domain specific effects that warrant careful consideration. Notably, the current NOAEL assessment is based on systemic endpoints such as body weight, organ pathology, and reproduction, which may be less sensitive to subtle neurobehavioral or microbiota alterations. Functional endpoints of the gut–brain axis, as assessed here, could therefore reveal effects at exposures below traditional safety thresholds, emphasizing the importance of incorporating such measures into risk assessment frameworks. The successful transfer of behavioral phenotypes via microbiota transplantation underscores shifts in the gut microbial community as a potential mechanism underlying social behavior impairments, independent of direct gene expression shifts. The ecological psychiatry approach emphasizes the significance of understanding how environmental factors like herbicides impact mental health at a population level in mice through complex microbiota-host interactions. Overall, our results advocate for a microbiota-gut-brain axis perspective in assessing pesticide toxicity, emphasizing that even regulatory-relevant exposures can impact behavior via microbial community alterations, and underscore the critical need for longitudinal studies to fully uncover the biological mechanisms behind glyphosate-induced behavioral changes and their ecological consequences.

## Supplementary information

Supplemental Information - Methods

Supplemental Information - Figures

## Data Availability

Metagenomic and transcriptomic data have been deposited at the European Nucleotide Archive and can be accessed with accession codes PRJEB111310 and PRJEB111812, respectively.
