# Supplementary material for: Towards ecological psychiatry: The herbicide glyphosate disrupts behavior through microbiota-gut-brain axis
Source: Mol Psychiatry. 2026 Jun 30;31(10):6046–55. doi: 10.1038/s41380-026-03693-2 (PMC13569421; doi:10.1038/s41380-026-03693-2)
Supplement: Supplementary file 1 [file 41380_2026_3693_MOESM1_ESM.docx]

Supplementary Methods

**Animals**

Mice were habituated in the animal facility for a minimum of a week prior to the commencement of glyphosate exposure. Sample size was determined based on power calculations focused on behavioral outcomes, which represented the primary endpoint of the study. Effect size estimates were informed by previous in-house data, and published studies. Using the Software G*Power 3.1, a minimum sample size of 10 animals per group was calculated to achieve 80% power at α = 0.05. Animals were housed under a 12 h light/dark cycle, temperature of 21 ± 1 °C and humidity of 55 ± 10 % in a standard mouse cage with *ad libitum* food (Teklad Global 18% Protein Rodent Diet). Mice were group housed per treatment, not more than 4 per cage, unless fighting occurred for males in which case they were housed individually. Body weight and water intake were monitored throughout the experiment.

**Behavioral Test Batteries**

A battery of behavioral tests was selected to assess multiple behavioral domains previously reported to be affected by glyphosate and other pesticide exposures, including exploratory activity, anxiety-like behavior, and social response (1–3). To minimize stress-related carryover effects and interference between assays, behavioral testing was conducted in a fixed order from least to most stressful as shown in Figure 1A & 4A. All behavioral tests were performed between 9 am to 12 pm, with at least one hour of habituation in the behavioral room prior to the tests (where required). The whole duration of the test was recorded using a ceiling-mounted camera. For the microbiota transplantation study, the marble burying test and novel object recognition test were not included as these domains were not impacted in the initial study in males.

**Three Chamber Test**

This test assesses social cognition such as social preference and social novelty preference. A white plastic rectangular arena with 2 removable access panels was used as a setup (center chamber: 13.5 × 20 × 20 cm, side chambers: 9 × 20 × 20 cm). The dividing panels had small circular openings (5 cm in diameter) allowing the mouse to explore all three chambers. Wire cylinder-shaped cages (10cm diameter, 13 cm height) were placed symmetric in the side chambers for the mice to interact but not fight with inanimate objects or conspecific mice. The test involved 3 x 10-minutes phases: habituation, social preference and social novelty preference phase. For the first phase, a mouse was placed into the middle chamber and explored the entire arena with empty wire cages. During both phase transitions, the mouse remained in the center chamber with panels closed to pause on exploration. For the second phase, sociability phase, the mouse explored the entire arena with one chamber containing an inanimate object and another chamber containing sex-, aged-matched conspecific mouse (familiar mouse). For the last phase, the social novelty phase, the mouse explored the entire arena with the inanimate object switched with sex-, aged-matched conspecific mice (novel mice). The cage with familiar mouse remained untouched. The amount of time spent exploring each chamber, by tracking the center of the mouse, was evaluated. Deeplabcut (Version 2.3.4) and SimBA (Version 1.77.2) were used to perform behavioral analysis (4–6) following the recommendations as described in <https://github.com/DeepLabCut/DeepLabCut> and <https://github.com/sgoldenlab/simba>.

**Elevated Plus Maze Test**

This test assesses anxiety-like behavior, based on the time mice spent on the open and closed arms of the plus maze. For the setup, grey plastic cross-shaped maze, comprised of two open and two closed arms (30cm long per each arm), were elevated 1 m above the ground. The test was conducted under white light (at an intensity of 100 lux) except for male cohort which was conducted under red light. Lighting conditions were adjusted to optimize detection of anxiogenic responses, and were empirically validated to ensure they did not influence behavioral outcomes. A mouse was placed onto the center of the maze facing an open arm and explored freely for 5 minutes. Entry into an arm was defined as all four paws crossing into the respective arm, which was defined by 85% of animal’s body being in an arm. ANY-Maze (Version 7.1, Stoelting Co) was used to perform behavioral analysis.

**Open Field Test**

This test assesses locomotion and anxiety-like behavior, based on the time mice spent in the center. For the setup, a grey plastic square box (45 × 45 cm) was used. The test was conducted under white light (at an intensity of 15 lux). A mouse was gently placed facing one of the walls and explored freely for 10 minutes. As previously described, by tracking the center of the mouse, the exploration behavior was scored using DeepLabCut and SimBA.

**Novel Object Recognition Test**

This test assesses hippocampal dependent short-term recognition memory, based on the time mice spent with familiar and novel inanimate objects. The same box used in open field test was used. This test was conducted over two days with 3 trials total, day 1 as habituation and day 2 as the test day with 2 trials. The open field test was served as day 1 habituation day. On the next day, the mouse went through 2 trials: familiarization and novelty phase. The trials were separated by 3 hours incubation time. For the familiarization phase, two identical inanimate objects were placed on adjacent corners approximately 5 cm from each wall. For the novelty phase one of the objects was switched with a novel object. In each trial, the mouse explored the arena for ten minutes. As previously described, by tracking the center of the mouse, the exploration behavior was scored using DeepLabCut and SimBA. However, the novel object recognition test results are not shown due to location-based bias observed during the habituation phase.

**Marble Burying Test**

This test assesses compulsive, stereotypic and anxiety-like behaviors in mice. A mouse was placed in a cage (35 × 28 × 18 cm, Length× Width × Height), filled with sawdust bedding (5 cm height) and 20 marbles on top of it (5 × 4 rows of marbles regularly spaced). The test duration was 30 minutes for males, 15 minutes for females to avoid the ceiling effect. The number of buried marbles was defined as sawdust covering 2/3 of the marble. The number was confirmed by 2 researchers blinded to experimental treatments.

**Stress Coping Test**

This test assesses the stress coping behavior. A mouse was placed in a clear glass cylinder (22 cm diameter) filled with 15-cm-depth water (25 ± 1 °C). To avoid odor transfer, the water was changed between each animal tested. The experimental mice were placed in the water for 6 minutes and the immobility(s) duration of the final 4 minutes was manually measured by a researcher blinded to the experimental treatments. The immobility was defined as a total absence of movement except slight motions to maintain the head above the water.

**Sample Collection**

Feces were collected at three time points: prior to glyphosate administration, before behavior commencement and after behavioral battery completion before cull. All fecal collections were conducted in the morning. For this, mice were placed under a sterilized standard feeder without bedding until defecation. For future microbiome analysis, fecal samples were frozen at -80 °C until DNA extraction.

For all cohorts, mice were culled via decapitation. Whole blood was collected in VACUETTE tube coated with K3E K3EDTA and centrifuged at 1500 × g at 4°C for 10 minutes to collect the plasma. For donor mice of the microbiota transplantation study, cecum was immediately transferred to anaerobic chamber for microbiota transplant inoculum preparation. All other samples were collected snap-frozen using dry ice and stored at -80 °C until further analysis.

**Corticosterone Measurement**

To collect the blood, a diagonal incision of 2mm at the tail tip was made. Approximately 100 μl of blood was collected in EDTA coated capillary tube to avoid blood coagulation. The collected blood was centrifuged at 1500 × g at 4°C for 10 minutes and aspirated plasma was frozen at -80°C until ELISA analysis.

**Transcriptomic Analysis**

RNA extracted from left amygdala was used for the analysis. The mRNA sequencing was conducted by AZENTA (Genewiz). There, RNA samples were quantified using Qubit 2.0 Fluorometer (Life Technologies, Carlsbad, CA, USA) and RNA integrity was checked with RNA Kit on Agilent 5600 Fragment Analyzer (Agilent Technologies, Palo Alto, CA, USA). The RNA sequencing library was prepared using the NEBNext Ultra II RNA Library Prep Kit for Illumina using manufacturer’s instructions (New England Biolabs, Ipswich, MA, USA). Briefly, mRNAs were initially enriched with Oligod(T) beads. Enriched mRNAs were fragmented for 15 minutes at 94 °C. First strand and second strand cDNA were subsequently synthesized. cDNA fragments were end repaired and adenylated at 3’ends, and universal adapters were ligated to cDNA fragments, followed by index addition and library enrichment by PCR with limited cycles. The sequencing libraries were validated using DNA Kit on the Agilent 5600 Fragment Analyzer (Agilent Technologies, Palo Alto, CA, USA), and quantified by using Qubit 2.0 Fluorometer (Invitrogen, Carlsbad, CA).

The sequencing libraries were multiplexed and clustered onto a flow-cell on the Illumina NovaSeq 6000 instrument according to manufacturer’s instructions. The samples were sequenced using a 2x150bp Paired End configuration v1.5. Image analysis and base calling were conducted by the NovaSeq Control Software v1.8.1 on the NovaSeq instrument. Raw sequence data (.bcl files) generated from Illumina NovaSeq was converted into fastq files and de-multiplexed using Illumina bcl2fastq 2.20 software. One mis-match was allowed for index sequence identification.

Reference annotation of *Mus musculus* (organism), GRCm38, UCSC, genome browser (GRCm38.p6), Ensemble (7) was used to perform reference genome of obtained sequences. Quality of outputs were assessed using FastQC using default parameters. Next, genes were annotated and counted using kallisto with default parameters (8). Further, transcriptomic analysis was undertaken in Rstudio (version 4.2.2). First, principal component analysis (PCA) was performed by permutational multivariate analysis of variance (PERMANOVA) from the function adonis2 from the vegan package using centered log-ratio (CLR) transformed values. To avoid zeroes in the data, they were replaced using “const” approach (9).

Target differential gene expression analysis was conducted by filtering the countable for genes encoding for social behavior (GO:0035176) and learning and memory (GO:0007611) using the Gene Ontology database (10). For this, differential expression was evaluated between each treatment group to the control group. Different abundance analysis was performed by fitting linear models in the CLR-transformed values. Due to the targeted approach, false discovery rate (FDR) correction was not applied to this analysis.

**Genomic sequencing**

Sequencing was outsourced to Teagasc Next Generation DNA sequencing Facility (Ireland). There, DNA was quantified, and shotgun metagenomics libraries were prepared using the Illumina DNA preparation kit (Illumina) with standard DNA input guidelines. Individual libraries were barcoded using Unique Duel Indices (UDIs) provided by Integrated DNA Technologies Ltd (IDT). Final libraries were quantified using a qubit high sensitivity kit (Life Technologies) and the average size of the fragments for each sample was determined on a Bioanalyzer using an Agilent High Sensitivity kit (Agilent). The molarity for each sample was determined and samples pooled equimolarly. The final pool was quantified using the Qubit High Sensitivity assay (Life Technologies) and sequenced on the Illumina NovaSeq 6000 using a 300 cycle SP v1.5 sequencing kit according to manufacturer’s instructions (Illumina).

For 16S sequencing, first, DNA was diluted to 5ng/ul and 16S metagenomics libraries were prepared as described in the Illumina 16S metagenomics guide (Forward primer: (5’-TCGTCGGCAGCGTCAGATGTGTATAAGAGACAGCCTACGGGNGGCWGCA-3’), reverse primer: (5’-GTCTCGTGGGCTCGGAGATGTGTATAAGAGACAGGACTACHVGGGTATCTAATCC-3’). Final libraries were quantified and pooled equimolarly and the concentration of the final pool determined using the Qubit high sensitivity assay (Life Technologies). The final pool was also run on an Agilent Bioanalyser high Sensitivity Chip (Agilent) to assess quality of the final pool. The pool was then diluted, combined with PhiX (30%) and sequenced on a MiSeq using a 600 cycle V3 reagent kit according to manufacturer’s instructions (Illumina).

**Gut microbiome profiling using whole genome sequencing**

The software, its versions and parameters used are accessible in detail in GitHub (<https://github.com/Benjamin-Valderrama/smooth_wgs_woltka>). Briefly, FASTQ files were quality checked using Kneaddata v0.12.0 (<https://github.com/biobakery/kneaddata>). Bowtie2 v2.5.1 (11) was used to decontaminate samples from reads aligning to the mouse host using the GRCm39 mouse genome as a reference (NCBI RefSeq assembly: GCF_000001635.27). Then, Bowtie2 v2.5.1 was again used to align the remaining reads to bacterial genomes available in the web of life (WoL) database (release 1). Woltka (12) v0.1.3 was used to generate the taxonomic and functional profiles of the communities. The functional profile of the analysed samples was then analysed with Omixer-rpmR (13) to annotate Gut-Brain Modules (GBMs)	(14).

**Gut microbiome profiling using 16S sequencing**

In brief, FASTQ files with raw sequences were loaded into R (version 4.1.1), where the Divisive Amplicon Denoising Algorithm (DADA2) version 1.22.0 (15) was used to construct a count table of amplicon sequence variants (ASV). For each step, default parameters were used except for the function filterAndTrim, where the argument maxEE was set as 2, truncQ as 2 and trimLeft as 30, truncLen as 150. SILVA database (v 138) (16) was used as a reference for taxonomic assignment up to the genus level, and PICRUSt2 (version 2.4.1) (17) was used with its default parameters to infer the genomic content from 16S data in terms of Enzyme Commission (EC) numbers in stratified and unstratified mode. The functional profile of the analyzed samples was then analyzed with Omixer-rpmR (13) to annotate GBMs (14).

**Gut microbiome analysis**

Downstream statistical analysis was performed with R (version 4.2.0) and RStudio (version 2022.7.1.554). Alpha diversity metrices were calculated using the alpha function from the microbiome package (version 1.24.0). Statistical differences of alpha diversity metrics between experimental groups were calculated using linear models. CLR transformation was performed as implemented in the function decostand, from the vegan (version 2.6.4) package with a pseudocount of 2/3. PCA was performed using the prcomp function and Aitchinson distances (i.e, Euclidean distance on CLR transformed data), as suggested previously in the literature (18). To test differences in beta diversity between groups, a PERMANOVA test was used as implemented in the adonis2 function from the vegan package, where 10 000 permutations and Euclidian distances were used. Differential abundance analysis of taxa and GBMs was performed by fitting general linear models on the CLR-transformed count tables. This analysis was performed by comparing each glyphosate treatment group to the control. No direct statistical comparisons between different doses were conducted. To account for multiple testing in analyses of microbiome features, the Benjamini-Hochberg post hoc method was applied (19). Statistically significant differences were determined using a value of 0.1 as a threshold. Plots were made using ggplot2 (version 3.4.4) and patchwork (version 1.1.3).

**Code and data availability**

R scripts and functions used for transcriptomic analysis can be found online at <https://github.com/thomazbastiaanssen/Tjazi>. Similarly, all related codes used for whole genome and 16S sequencing is available online at <https://zenodo.org/records/18633858>. Metagenomic and transcriptomic data have been deposited at the European Nucleotide Archive and can be accessed with accession codes PRJEB111310 and PRJEB111812, respectively.

Supplementary Reference

1.	Aitbali Y, Ba-Mhamed S, Bennis M. Behavioral and Immunohistochemical Study of the Effects of Subchronic and Chronic Exposure to Glyphosate in Mice. Front Behav Neurosci. 2017 Aug 8;11:146.

2.	Del Castilo I, Neumann AS, Lemos FS, De Bastiani MA, Oliveira FL, Zimmer ER, et al. Lifelong Exposure to a Low-Dose of the Glyphosate-Based Herbicide RoundUp® Causes Intestinal Damage, Gut Dysbiosis, and Behavioral Changes in Mice. Int J Mol Sci. 2022 May 17;23(10):5583.

3.	Pu Y, Yang J, Chang L, Qu Y, Wang S, Zhang K, et al. Maternal glyphosate exposure causes autism-like behaviors in offspring through increased expression of soluble epoxide hydrolase. Proceedings of the National Academy of Sciences. 2020 May 26;117(21):11753–9.

4.	Mathis A, Mamidanna P, Cury KM, Abe T, Murthy VN, Mathis MW, et al. DeepLabCut: markerless pose estimation of user-defined body parts with deep learning. Nat Neurosci. 2018 Sept;21(9):1281–9.

5.	Nath T, Mathis A, Chen AC, Patel A, Bethge M, Mathis MW. Using DeepLabCut for 3D markerless pose estimation across species and behaviors. Nat Protoc. 2019 July;14(7):2152–76.

6.	Nilsson SR, Goodwin NL, Choong JJ, Hwang S, Wright HR, Norville ZC, et al. Simple Behavioral Analysis (SimBA) – an open source toolkit for computer classification of complex social behaviors in experimental animals [Internet]. bioRxiv; 2020 [cited 2024 May 28]. p. 2020.04.19.049452. Available from: https://www.biorxiv.org/content/10.1101/2020.04.19.049452v2

7.	Okazaki Y, Furuno M, Kasukawa T, Adachi J, Bono H, Kondo S, et al. Analysis of the mouse transcriptome based on functional annotation of 60,770 full-length cDNAs. Nature. 2002;420(6915):563–73.

8.	Bray NL, Pimentel H, Melsted P, Pachter L. Near-optimal probabilistic RNA-seq quantification. Nat Biotechnol. 2016 May;34(5):525–7.

9.	Lubbe S, Filzmoser P, Templ M. Comparison of zero replacement strategies for compositional data with large numbers of zeros. Chemometrics and Intelligent Laboratory Systems. 2021 Mar 15;210:104248.

10.	Ashburner M, Ball CA, Blake JA, Botstein D, Butler H, Cherry JM, et al. Gene Ontology: tool for the unification of biology. Nat Genet. 2000 May;25(1):25–9.

11.	Langmead B, Salzberg SL. Fast gapped-read alignment with Bowtie 2. Nat Methods. 2012 Apr;9(4):357–9.

12.	Zhu Q, Huang S, Gonzalez A, McGrath I, McDonald D, Haiminen N, et al. Phylogeny-Aware Analysis of Metagenome Community Ecology Based on Matched Reference Genomes while Bypassing Taxonomy. mSystems. 2022 Apr 4;7(2):e00167-22.

13.	Darzi Y, Falony G, Vieira-Silva S, Raes J. Towards biome-specific analysis of meta-omics data. ISME J. 2016 May;10(5):1025–8.

14.	Valles-Colomer M, Falony G, Darzi Y, Tigchelaar EF, Wang J, Tito RY, et al. The neuroactive potential of the human gut microbiota in quality of life and depression. Nat Microbiol. 2019 Apr;4(4):623–32.

15.	Callahan BJ, McMurdie PJ, Rosen MJ, Han AW, Johnson AJA, Holmes SP. DADA2: High-resolution sample inference from Illumina amplicon data. Nat Methods. 2016 July;13(7):581–3.

16.	Quast C, Pruesse E, Yilmaz P, Gerken J, Schweer T, Yarza P, et al. The SILVA ribosomal RNA gene database project: improved data processing and web-based tools. Nucleic Acids Res. 2013 Jan;41(Database issue):D590-596.

17.	Douglas GM, Maffei VJ, Zaneveld JR, Yurgel SN, Brown JR, Taylor CM, et al. PICRUSt2 for prediction of metagenome functions. Nat Biotechnol. 2020 June;38(6):685–8.

18.	Gloor GB, Macklaim JM, Pawlowsky-Glahn V, Egozcue JJ. Microbiome Datasets Are Compositional: And This Is Not Optional. Front Microbiol [Internet]. 2017 Nov 15 [cited 2024 Nov 1];8. Available from: https://www.frontiersin.org/journals/microbiology/articles/10.3389/fmicb.2017.02224/full

19.	Benjamini Y, Hochberg Y. Controlling the False Discovery Rate: A Practical and Powerful Approach to Multiple Testing. Journal of the Royal Statistical Society: Series B (Methodological). 1995;57(1):289–300.
