# Supplementary material for: Towards ecological psychiatry: The herbicide glyphosate disrupts behavior through microbiota-gut-brain axis
Source: Mol Psychiatry. 2026 Jun 30;31(10):6046–55. doi: 10.1038/s41380-026-03693-2 (PMC13569421; doi:10.1038/s41380-026-03693-2)
Supplement: Supplementary file 2 [file 41380_2026_3693_MOESM2_ESM.docx]

**Supplementary Figures**

**Figure S1.** Glyphosate was overall well tolerated throughout the study by both male (blue) and female (red) mice. (A) Weight change (B) water intake levels (Overall: *F*_3,9_ = 5.43, *p* = 0.021; High: * *p* = 0.023). Repeated general linear model followed by pairwise comparison; n = 10-11 per treatment group per sex; data represent mean ± SEM.

**Figure S2.** Glyphosate exposure decreased spleen size in males (blue) but not in females (red). (A) Adrenal glands (B) Spleen (Overall: *F*_3,9_ = 5.43, *p* = 0.021; High: * *p* = 0.008) (C) Cecum (D) Colon Length. * p ≤ 0.05; one-way ANOVA followed by post-hoc Tukey analysis; n = 10-11 per treatment group per sex; data represent mean ± SEM.

**Figure S3.** Plasma corticosterone levels after stress (stress coping test) over 120 minutes showed no differences in physiological stress responses across treatment groups for (A) males (blue, *F*_3,23_ = 1.41, *p* =0.265) and (B) females (red, *F*_3,27_ = 0.955, *p* =0.428). repeated general linear model; n = 10-11 per treatment group per sex; data represent mean ± SEM.

**Figure S4.** No behavioral impairments were observed in above test phases upon glyphosate exposure in males (blue) and females (red). (A) The three-chamber test sociability phase showed no effect of glyphosate exposure, as both sexes across all treatment groups spent significantly more time in the chamber with a conspecific mouse compared to an inanimate object (Two-way repeated-measures ANOVA). In the social novelty phase, only females in the medium-dose group spent significantly more time with a novel mouse over a familiar mouse. (B) In the elevated plus maze, which assesses anxiety-like behavior, no significant differences were found across treatment groups for either sex (Kruskal-Wallis test, Male: *H*_3_ = 2.21, *p* = 0.53, *η^2^* < 0; One-way ANOVA, Female: *F*_3,42_ = 2.06, *p* = 0.121, *η^2^* = 0.137). (C) The marble burying test, used to measure repetitive behavior, showed no significant differences across treatment groups for either sex (Kruskal-Wallis test, Male: *H*_3_ = 0.57, *p* = 0.904, *η^2^* < 0; Female: *H*_3_ = 5.15, *p* = 0.161, *η^2^* = 0.06). A possible ceiling effect was observed in males, with nearly all marbles buried. (D) The stress coping test revealed no changes in response behavior across all treatment groups for both sexes (Kruskal-wallis test, Male: *H*_3_ = 5.39, *p* = 0.146, *η^2^* = 0.06; Female: *H*_3_ = 0.80, *p* = 0.849, *η^2^* < 0). * *p* ≤ 0.05; one-way ANOVA followed by post-hoc analysis; n = 10-11 per treatment group per sex; data represent mean ± SEM.

**Figure S5.** Alpha diversity of gut microbiota showed no difference upon glyphosate exposure on (A) Chao1 (B) Shannon (C) Simpson index for males (blue) and females (red). *p* = *n.s.*; linear model; n = 10-11 per treatment group per sex

**Figure S6.** Gut permeability related gene expression showed no changes upon glyphosate exposure in (A, blue) male and (B, red) female mice in ileum and colon. *p* = *n.s.*; One-way ANOVA; n = 10-11 per treatment group per sex; data represent mean ± SEM.

**Figure S7.** Amygdala transcriptomic changes were observed on genes related to social behavior, learning and memory upon glyphosate exposure in males. (A) Principal component analysis (Ellipse denotes 95% CI) (B)Volcano plot of social behavior and learning related genes at high dose glyphosate exposure (General linear model) (C) Transcriptomic analysis of the male amygdala identified genes affected by glyphosate exposure per dose related to social behavior (*Nr2e1*), learning and memory (*Abcc8, Arc, B2m, Chst10, Cic, Dbi, Gm2a, Klk8, Paip2, Pak6, Reln, Tlr2, Ttbk1, Vdac3*), or both (*Grin1, Htt*) * *p* ≤ 0.05; General linear model; n = 10-11 per treatment group

**Figure S8.** Cecal microbiota transplantation in males presented some microbiota-independent alterations in the recipient mice. (A,B) Recipient mice were treated with an antibiotic cocktail for one week to deplete original microbiota, leading to an expected initial weight loss, which recovered prior to cecal microbiota transplantation via oral gavage. The microbiota transplantation procedure and subsequent behavioral assessments were well tolerated, with no significant differences in weight gain patterns or organ sizes observed between groups throughout the experiment (*p* = *n.s.*). (C) During the sociability phase of the three-chamber test, both recipient groups spent significantly more time in the chamber with a conspecific mouse than with an inanimate object. (D) Anxiety-like behavior, assessed by the elevated plus maze, showed no differences between treatment groups (*t*_1,20_ = -0.33, *p* =0.745). (E) Stress-coping behavior also showed no significant differences between groups (*U* = 52, *Z* = -0.528, *p* = 0.598). (F) Post-stress coping test corticosterone levels showed no significant differences in physiological stress response between treatment groups (*F*_1,20_ = 1.246, *p* = 0.278). (G) Genes upregulated or downregulated in the original male study were tested in recipients to assess if microbiota transplantation would reproduce this genetic phenotype. However, no such gene expression changes were observed in the recipients (*p* = n.s.). T-test (except for A&F using repeated general linear model, C using Two-way repeated-measures ANOVA); n = 11-12 per treatment group; data represent mean ± SEM.

**Figure S9.** No alterations in alpha diversity of gut microbiota in males (A) Chao1 (B) Shannon (C) Simpson index for donor (orange) and recipient (green). * *p* = *n.s.*; linear model; n = 11-12 per treatment group.
